# Drivers of international convergence in digital two-sided platform markets: The case of dominant business model components in online lending

**DOI:** 10.1371/journal.pone.0342410

**Published:** 2026-07-20

**Authors:** Sabine Pur, Stefan Huesig, Christoph Schmidhammer

**Affiliations:** 1 Management, IU International University of Applied Sciences, Erfurt, Germany; 2 Innovation Research and Management of Technology, Chemnitz University of Technology, Chemnitz, Germany; 3 Bank Management and Financial Econometrics, Deutsche Bundesbank University of Applied Sciences, Hachenburg, Germany; University of Jaen: Universidad de Jaen, SPAIN

## Abstract

Online lending marketplaces have emerged as potentially disruptive players in the financial industry by digitally connecting borrowers and lenders. However, little is known about the emergence of dominant business model patterns in this regulated, platform-based service industry. Drawing on dominant design theory, this study examines the international convergence of business model components in online lending since 2005. Using a comparative, longitudinal case study of the market leaders Funding Circle (the UK), LendingClub (the USA), and auxmoney (Germany), we analyze the Business Model Canvas components “customer segments” and “value propositions.” Our findings reveal a convergence toward peer-to-institution models, shaped by regulatory pressures, technological advances, and institutional capital. All marketplaces now address underserved borrower segments such as SMEs, the self-employed, and founders, while excluding retail investors. This indicates the emergence of internationally dominant business model components in potentially disruptive two-sided platform markets. We synthesize these findings into a generic, process-oriented model explaining how platform convergence unfolds across national contexts. This study extends dominant design theory to business models in regulated service industries and highlights external forces as key drivers of standardization. It offers practical insights for policymakers, institutional investors, and marketplace operators navigating scalability, regulation, and financial integration.

## Introduction

The financial industry has undergone significant transformations since the 2008 banking crisis, driven by advancing digitization, networking, and business model innovations with disruptive potential [1–3]. This is particularly evident in the rise of financial technology companies (FinTechs), which challenge traditional financial services through technology-driven business models [4,5]. Online marketplace lending has become one of the fastest-growing segments among various overlapping FinTech subcategories, such as crowdsourcing, mobile payments, and cryptocurrencies. These platforms connect lenders and borrowers, manage repayment obligations, and disrupt the traditional role of banks as financial intermediaries [4,5].

The first online lending marketplace, Zopa, launched in the UK in 2005, followed by Prosper (2006), LendingClub (2007) in the USA, smava and auxmoney (2007) in Germany, and many others worldwide. Since then, marketplace lending has rapidly expanded and has become one of the most prevalent FinTech business models in the UK, the USA, Europe, and China, with strong growth in India, Japan, South Korea, and other fast-growing economies [4–6]. Peer-to-peer (p2p) lending, the idea of individuals lending money to one another, has existed for centuries in informal settings such as the Friendly Societies in the UK [7] and neighborhood circles in France [8], aimed to promote financial inclusion and empowerment [7]. Modern online lending marketplaces build on this legacy by offering financing to individuals and businesses excluded from traditional banking while enabling financial and social returns for investors [2,7]. Over time, however, most marketplaces have shifted from retail investors to institutional capital, with regulatory changes and the professionalization of the sector contributing to this shift [9–11].

New markets and technologies often begin with different business models as companies experiment with diverse strategies to meet emerging customer needs [12,13]. Over time, as customer preferences become clearer, the various market players adjust their business models accordingly to fit customer expectations and the value network better [14–18]. As part of this process, external drivers such as regulatory changes, technological advances, and market dynamics play an important role in shaping business models [19–21], although their specific influence has received limited attention so far [22]. Eventually, a temporal ideal business model emerges, perceived as the de facto standard within the industry [14,23]. This concept, widely known as the “dominant design,” was originally developed for product and technological innovation [24].

While dominant design theory is well established from an architectural or technology-oriented perspective [25,26], its application to business models remains underexplored, particularly evident in regulated service industries and digital two-sided platform markets [10,27,28]. Understanding these dynamics is crucial, as it enables companies in regulated platform markets such as financial services to adapt their business models to changing customer needs, regulatory requirements, and market structures in a targeted manner.

Prior research on online lending marketplaces has often focused on information asymmetries, signaling, voluntary disclosure, investor behavior, geographical frictions, and platform monitoring. Friendship networks may serve as signals of borrower quality, while voluntary disclosure can increase funding success but may also be associated with higher default risk and potential information manipulation [29,30]. Other studies show that geographical and behavioral frictions persist in digital lending markets, expert investors appear to make better use of available listing information than nonexpert investors, and regulatory monitoring can affect platform stability [31–34]. While this literature provides important insights into lending and investment decisions as well as platform risks, less is known about how these mechanisms translate into business model development and convergence at the platform and industry level. A business model perspective can therefore complement existing research by examining how such mechanisms are reflected in structural business model adaptations of online lending marketplaces, particularly in the shift toward institutional investors.

To address these research gaps, this study examines whether an internationally dominant business model design has emerged since the rise of online lending marketplaces in 2005. Building on the framework of the dominant design theory, we analyze the business model development of the leading online lending marketplaces in the UK (Funding Circle), the USA (LendingClub), and Germany (auxmoney). Our focus is on the building blocks of “customer segments” and “value propositions” from the Business Model Canvas [35], as these are considered critical indicators of product-market fit [36] and are used in the analysis of business model convergence [10].

We thus pursue the following research question: How and why do the business models of leading online lending marketplaces differ or converge toward a dominant design internationally or in their home markets?

To address this question, we conducted a longitudinal, comparative multiple-case study, following the methodological framework of Yin [37] and Eisenhardt and Graebner [38]. We uncover the external factors and strategic choices that drive convergence or divergence by analyzing these marketplaces’ historical development and current configurations. The selected countries represent mature online lending markets and share important institutional similarities while differing in terms of regulatory details and investor structures.

Our results show a convergence of business models in the key components of customer segments and value propositions among market leaders in the UK, the USA, and Germany.

This paper contributes to research on dominant designs and business model innovation by transferring the dominant design concept to platform-based business models and proposing a generic process model for the emergence of dominant components in potentially disruptive two-sided platform markets. This model offers a basis for understanding the development of dominant components and anticipating future trajectories of platform-based business models. It highlights the role of external pressures, such as regulation, technological change, and investor dynamics, as underexplored drivers of business model convergence, and shows how these forces can foster upmarket shifts toward higher customer segments. Beyond its theoretical contribution, this study also offers actionable insights for stakeholders navigating platform evolution in regulated financial markets.

The remainder of this paper is organized as follows: Section 2 outlines the theoretical foundations. Section 3 presents the research design, including the case study approach, data sources, and analysis. Sections 4 and 5 provide the case findings and cross-case comparison. Section 6 develops the theoretical contributions, discusses the practical implications, and introduces the generic model. Section 7 concludes with limitations and future research directions.

## Theoretical background and conceptual foundations

### Business model innovation and the emergence of dominant designs

Since the mid-1990s, researchers have been discussing the business model concept, with most agreeing that it describes how an organization creates value and generates revenue [39]. This paper adopts the definition of Osterwalder and Pigneur [35], who describe a business model as “the rationale of how an organization creates, delivers, and captures value.”

To remain competitive, companies must continuously adapt their business models. Accordingly, research on business model innovation has expanded [25,27,40]. However, scholars differ on how much change qualifies as business model innovation. Foss and Saebi [40] emphasize this ambiguity, while Ramdani et al. [22] argue that business model innovation can range from minor adjustments to complete structural reconfigurations. These changes typically affect four domains: value proposition, operational value, human capital, and financial value. Schallmo et al. [41] highlight that the degree of novelty in business model innovation is primarily assessed from the customer’s perspective and results from novel combinations of business model components that create new value for customers and partners.

External factors, such as technological advancements, regulatory requirements, and shifts in consumer behavior, are frequently cited as key drivers of business model innovation [19–21]. Although their importance is widely acknowledged, their precise influence, particularly in regulated two-sided platform markets such as online lending, remains underexplored [22].

When new technologies or markets emerge, customer needs are often unclear, and multiple business models compete to address nascent demands [12,13,16,42]. Companies respond with radical innovations and adapt their business models iteratively to changing customer preferences and value networks [14–18]. Over time, a temporarily optimal configuration emerges that aligns with the needs of most market participants and is perceived as the industry standard [14,23]. Following Utterback and Abernathy [24], we refer to this standard as the “dominant design.”

The emergence of a dominant design marks a turning point for the industry [23,25]. From this moment on, industry dynamics shift as innovation increasingly focuses on processes rather than products [23,43–46]. Dominant designs influence technology life cycles and strategic priorities [25,47], often leading to more efficient product development and a reduced number of competitors [24,48]. Moreover, Brem et al. [25] empirically demonstrated a negative relationship between the emergence of a dominant design and the overall degree of innovation within an industry.

When applied to business models, the concept of a dominant design implies that successful companies adopt similar structural components that serve as the de facto standard within an industry. Companies that deviate from this configuration typically operate in niche markets [13,14,23].

Despite its relevance, the concept of dominant design has primarily been explored in technological or architectural contexts [25,26]. Its application to business models, especially in two-sided platform markets and regulated service industries, remains underdeveloped [10,27,28]. While recent studies examine standardization in digital innovation ecosystems, they focus mainly on technological, regulatory, or organizational aspects [28]. The role of business model structures in this context, particularly within two-sided platform markets, remains insufficiently understood.

This gap is particularly relevant for two-sided platform markets in the financial services sector, such as online lending marketplaces, where regulatory frameworks, technological change, and institutional investors’ strategic behavior contribute to business model evolution, potentially leading to dominant configurations.

### Influence of disruptive innovations on business models

Disruptive innovations are new offerings that initially fall below established market criteria but open up alternative ways to create value by targeting underserved or previously excluded customer segments [49,50]. Over time, these innovations improve and can redefine industry standards and eventually displace established solutions [51]. A key element of this process is the development of novel business models that enable market participants to operate outside traditional structures and attract new demand [52,53].

This evolution often follows a strategic pattern described in disruptive innovation theory, where entrants begin by serving marginalized market segments but subsequently migrate upmarket by improving their offerings and focusing on more profitable and demanding customer groups [50].

Online lending marketplaces are an example of this development in the financial sector. They initially bypassed traditional banks to serve borrower groups with limited access to credit before evolving into professionally managed marketplaces that integrate institutional capital and challenge established lending models [2,54,55].

### Business models in two-sided platform markets

Two-sided platform markets are a distinct type of business model in which a platform mediates between two user groups characterized by mutual attraction and network effects [2,56]. A prominent example is online lending marketplaces, which connect borrowers and lenders. While borrowers seek low interest rates, lenders aim for high returns, creating an inherent conflict of objectives [57].

Network effects in such platforms can be categorized into direct vs. indirect and same-side vs. cross-side. Direct network effects arise when increasing participation on one side raises value for the same side. Indirect effects occur when complementary products or services attract users [56,58,59]. Same-side effects boost the value of a group due to its own growth [56], while cross-side effects mean that growth on one side enhances attractiveness for the other [58,60]. These effects are interlinked, increasing management complexity [56,61]. For instance, more borrowers attract institutional investors, raising the need for sophisticated risk management.

A key challenge is the “chicken and egg problem,” where marketplaces have to attract users on both sides at the same time [2,57,62]. To overcome this challenge, platform operators often focus on the revenue-generating side and treat the other side as financially neutral or deliberately subsidize it to encourage participation [57,62,63]. Long-term success requires balancing user growth and monetization strategies [61].

Building on this, two-sided platform markets introduce unique structural dynamics, particularly network effects, that further influence business model evolution and convergence.

### Conceptual framework for analyzing business models and dominant designs

A widely used framework for developing and analyzing business models is the Business Model Canvas by Osterwalder and Pigneur [35], which conceptualizes business models across nine interconnected building blocks [64,65]. At its core is “value propositions,” surrounded by “customer relationships,” “channels,” and “customer segments” on one side, and “key resources,” “key activities,” and “key partners” on the other. These are complemented by “cost structure” and “revenue streams” [35].

Recent research identifies “customer segments” and “value propositions” as key to recognizing dominant designs in an industry. Pur et al. [10] showed this by analyzing three leading German online lending marketplaces. While operational elements such as key resources, key activities, key partners, and associated cost structures were structurally similar, the marketplaces diverged significantly in customer segments and value propositions due to strategic positioning in a common national market. All marketplaces began as providers of p2p lending but evolved to serve distinct customer segments with differentiated value propositions, resulting in different revenue streams. In contrast, customer relationships and channels remained largely standardized.

These findings indicate that customer segments and value propositions are central to understanding the convergence or divergence of business models within an industry. This aligns with Ramdani et al. [22], who highlighted these components as dimensions of business model innovation, and Schallmo et al.’s [41] customer-centric perspective. It also resonates with the Value Proposition Canvas by Osterwalder et al. [36], which focuses on these components to ensure a product-market fit.

Building on these insights, this study focuses on customer segments and value propositions as critical indicators for identifying internationally dominant configurations in online lending marketplaces.

## Research design

### Methodological approach

Our research design is based on the methodological principles of Strang [66] and combines a post-positivist and pragmatic perspective, enabling a systematic analysis of business model development while considering external influences. This integrated approach ensures methodological clarity and supports the structured assessment of convergence patterns in evolving market contexts. Within this framework, the level of analysis is at the company/organizational level, examining the development of the current leading online lending marketplaces from three key countries where the industry originated: the UK (Funding Circle), the USA (LendingClub), and Germany (auxmoney). These countries were selected because they provide a comparable context, with similarly developed economies and mature financial markets, that allows us to analyze structural similarities in business model evolution over time.

We apply a multiple/cross-case study approach based on Yin [37] and Eisenhardt and Graebner [38] to investigate these dynamics. This method is particularly suitable for our research as it enables an in-depth investigation of real business model developments in their contextual environment. Following Swanborn [67], we adopt a retrospective perspective and analyze multiple points in time to capture the evolution of business models. This longitudinal approach allows us to track key adaptations and convergence trends in the selected online lending marketplaces. By analyzing these developments across countries, we explore theoretical assumptions about dominant designs and business model innovation and provide a basis for applying these findings to other industries through further case studies or large-scale research.

### Data collection and reliability

The unit of analysis is the business model of the selected market leaders. We track their evolution and compare qualitative changes with originated loans from founding to March 2024. This allows us to assess how adjustments in customer segments and value propositions [35,36] correlate with market performance. We hypothesize that business model innovation will impact loan volumes positively or negatively and that market leaders’ business models will increasingly converge as they adapt to clearer and evolving customer needs.

To ensure validity and reliability, we employ data triangulation, which is a key principle in qualitative research [37,68]. Our analysis is based on 236 secondary sources, which can be divided into:

- Company publications, including annual reports, press releases, and blogs from the online lending marketplaces.
- External sources, such as entries in the Federal Gazette, industry reports, and published interviews with key players.

The selection criteria for these secondary sources include relevance, credibility, and timeliness to ensure the robustness of our dataset [66]. The data and information extracted from them are cited directly in the study to make the results more comprehensible. Given the transparency of online lending marketplaces, these publicly available sources enable systematic observation and ensure a robust empirical basis. In addition, inconsistencies between sources are cross-validated using multiple independent data sets and a comparative review framework [66].

### Data analysis

The analysis follows a two-stage approach. First, we conduct single/within-case analyses, examining each market leader in its national context to understand the evolution of its business model. Second, we conduct a cross-case analysis, contrasting similarities and differences to gain insights into the mechanisms driving business model convergence or divergence.

The empirical analysis focuses on the two customer-oriented building blocks of the Business Model Canvas “customer segments” and “value propositions,” which serve as the conceptual framework for analyzing the systematic development and convergence of business models [35].

To ensure transparency, we follow Strang [66] in our multiple/cross-case analysis, systematically mapping business model patterns to validate convergence mechanisms.

Based on this research design, we pursue the following overarching research question:

How and why do the business models of leading online lending marketplaces differ or converge toward a dominant design internationally or in their home markets?

The following sub-research questions guide our single/within-case analyses:

- How has the online lending marketplace industry developed in the respective country?
- How and why has the business model of the respective market leader developed over time?

To operationalize these research questions, we assess the prevailing business model components by combining qualitative case coding and loan volume trends, ensuring a structured and comparative approach to analyze the standardization of business models across different regulatory and market environments.

The findings from the single/within-case and cross-case analyses form the basis for deriving a generic model that explains the emergence of dominant business model components in potentially disruptive platform-based industries. To enhance clarity and practical applicability, this model adopts a process-oriented visualization inspired by the Business Process Model and Notation (BPMN) principles, providing a structured and intuitive representation of key processes and interactions [69].

## Single case studies

### Marketplace lending in the UK: Funding Circle

#### Institutional framework and lending landscape.

The UK features a centralized banking system with London as an international financial hub, hosting global institutions alongside regional and local banks [70]. Capital market-based financing and a strong venture capital sector, supported by insurance companies and pension funds, foster innovation [71]. Facing growing competition from digital challengers, traditional banks are modernizing their services, often through partnerships or acquisitions of FinTechs such as online lending marketplaces [72,73]. London also anchors a dynamic FinTech ecosystem that drives alternative lending models [73,74].

The Bank of England supervises banks and large financial institutions [75]. The Financial Conduct Authority (FCA) regulates financial markets and service providers, adheres to the standards of the Basel Committee on Banking Supervision (BCBS), and cooperates closely with European regulators [76].

In the UK, genuine p2p lending is well established, allowing direct loan agreements between borrowers and lenders without intermediary banks. In this model, marketplaces operate under brokerage contracts and generate revenue through commissions [77].

#### Market situation and key players.

The UK online lending marketplace industry comprises 122 providers with a market size of £397.7m (as of August 2023). Funding Circle leads with a 46.8% market share [73]. Market growth is expected to slow down due to consolidation, macroeconomic conditions, and tighter regulations [11].

Two major competitors exited the marketplace model: Zopa, which fully transitioned to Zopa Bank in 2021 [78], and Ratesetter, acquired by Metro Bank in 2020 [79]. In addition, platforms like Lending Works [80] and Assetz Capital [81] withdrew from the retail investment segment and now focus on institutional lending.

Key drivers of this shift include the impact of COVID-19, declining retail investor participation, and stricter FCA requirements for marketplace operations [82–84]. These requirements included, for example, a limitation on retail investment volumes [85]. These developments have made marketplace lending less attractive for marketplaces and retail investors although the revised rules are seen to enhance consumer protection [73]. A few niche providers like Folk2Folk still allow retail investments, targeting specific borrower segments such as property developers or farmers [86].

#### Business model development of Funding Circle.

Funding Circle was founded in 2010 to provide loans to small and medium-sized enterprises (SMEs) funded by retail investors [86,87]. The marketplace targeted a submarket traditionally underserved by UK banks [88]. The next year (2011), Index Ventures joined as the first institutional investor [89]. In 2013, the UK government began lending via the marketplace, and Funding Circle expanded into the U.S. market [90]. In 2014, it entered the German market [91]. In 2015, it merged with its much smaller German competitor Zencap, which had focused on the continental European business. At the time, large venture capital funds such as Index Ventures, Union Square, and Accel Partners were backing Funding Circle [92]. The merger with Zencap facilitated Funding Circle’s access to the German, Dutch, and Spanish markets, where Zencap was already represented [93]. In 2016, it discontinued its business in Spain and its real estate lending business, which was only available in the UK [94,95]. In 2019, Funding Circle expanded its institutional investor offerings to include a receivables-backed bond program and a UK economic impact fund [96]. In 2020, at the onset of COVID-19, Funding Circle paused its retail investment business [97] and secondary market offerings [98]. In the same year, it ceased operations in Germany [99] and the Netherlands due to cost concerns [100]. For German borrowers, it has since referred to the Kreditanstalt für Wiederaufbau’s (KfW) domestic funding [101], and for Dutch borrowers to the Chamber of Commerce and SME funding [102]. In 2021, Funding Circle introduced FlexiPay, a short-term loan product [103]. A year later (2022), it closed its investment business for retail investors [82]. In 2023, it hired the former LendingClub president to expand the U.S. market [104]. The same year, the marketplace no longer accepted applications under the Government Recovery Loan Scheme and referred to Self Employed Loans offered on the marketplace for a certain time [105]. However, it simultaneously partnered with Atom Bank to support SME financing under the revised Recovery Loan Scheme [106]. Additionally, it partnered with Tungsten and PayPoint to extend its reach to UK SMEs [107] and provided brokers with FlexiPay, enabling borrowers to pay by card or transfer [108,109]. Later in 2023, the Funding Circle loan portfolio was securitized by Waterfall Asset Management, allowing institutional investors to acquire further loans via the asset-backed securities (ABS) market [110]. At the end of 2023, the company announced that it had received an SBA Small Business Lending Company (SBL) license from the U.S. Small Business Administration, enabling it to offer SBA 7(a) small loans in the USA starting in 2024 [111]. 2024 began with a financing partnership with Barclays Bank and TPG Angelo Gordon [112]. Since its founding (as of March 2024), Funding Circle has mediated £16.9 billion to over 150,000 companies [113].

**Fig 1:**
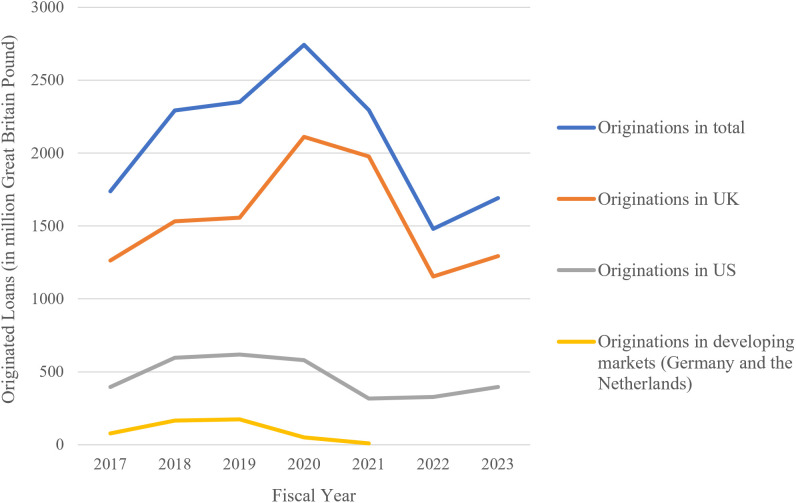
Annual development of originated loans at Funding Circle.

The business model development described above is accompanied by the following development of originated loans per fiscal year in Fig 1 (all originated loans from the document archive from Funding Circle [114]; loans include FlexiPay since 2021).

Funding Circle has focused on SMEs since it was founded. Institutional investors became involved one year after the company was founded (2011), followed by the British government in 2013. The company’s internationalization efforts remained limited: Funding Circle withdrew from continental Europe, and the volume of loans declined in the USA. New products for institutional investors in 2019 appear to have had a positive impact. The business model changed significantly in 2022 with the closure of the retail investor segment. This change became even more pronounced in 2023, marked by increasing loan volumes and new strategic initiatives, including broader institutional partnerships, new payment options, and the securitization of portfolios via the ABS market. The increase in U.S. lending could be due to increased internationalization efforts under the new U.S. leadership.

As of March 2024, Funding Circle exclusively mediates loans from institutional investors to SMEs, a category that now includes the self-employed, freelancers, and founders, although the exact timing of their inclusion is not documented. Loan types differ primarily in terms of amount (from £1,000 to £5 million), maturity (from 3 to 84 months), and interest rate (from 6.9% p.a.). The loan type and conditions depend on the intended use and creditworthiness.

Table 1 shows the current business model (as of March 2024) in the building blocks of customer segments and value propositions.

**Table 1:** Customer segments and value propositions at Funding Circle.

	Customer segments	Borrower				Lender
		SME				Institutional investors
**Value propositions**	**Loan type**	Business Loans^a^	Government-Backed Recovery Loans (Recovery Loan Scheme)	Asset Finance	FlexiPay	ND
	**Loan range** **(in £)**	10,000–500,000	25,001–250,000	10,000–5 m	1,000–250,000	ND
	**Term** **(in months)**	6–72	6–72	6–84	3	ND
	**Interest rates p.a. (in %)**	From 6.9	From 13.7	ND	0	ND
	**Fees**	One-off completion fee	One-off completion fee	ND	Flat 3% fee on each invoice	ND

^a^Various loans on the same terms, e.g., loans for the self-employed, unsecured loans, working capital loans, loans for limited liability companies

Note: ND = not disclosed; no reliable publicly available information was identified in the sources analyzed.

### Marketplace lending in the USA: LendingClub

#### Institutional framework and lending landscape.

The USA operates a decentralized central banking system, consisting of twelve regional Federal Reserve Banks under the central control of the Federal Reserve Board in Washington [115]. The financial market includes major global institutions alongside regional and local banks [116]. Capital and venture capital markets are highly developed, with pension funds and insurance companies as key investors [71]. Traditional banks are facing growing competition from FinTech start-ups, which is prompting them to modernize their digital services [116]. Many are now collaborating with, acquiring, or being acquired by FinTechs such as online lending marketplaces. Innovation hubs such as Silicon Valley and New York play a central role in promoting digital lending and alternative financing models [117].

Financial supervision is distributed across multiple national authorities. Key institutions include the Federal Reserve Bank of New York and the Board of Governors of the Federal Reserve System, both members of the BCBS [118].

In the USA, online lending marketplaces generally use a debt-dependent notes model, where a partner bank grants the loan, but the receivable is completely transferred to the marketplace. The marketplace then securitizes the repayment and interest claims by issuing corresponding bonds [77].

#### Market situation and key players.

The online lending marketplace industry in the USA comprises 13 active marketplaces with a total volume of approximately $1.5 billion (as of September 2023). LendingClub holds the largest market share at 20.8%, followed by Upstart Network (13.3%), On Deck Capital (12.1%), and Prosper (5.3%) [117].

While LendingClub has evolved into a full-spectrum FinTech marketplace bank in 2021 [119], competitors have pursued narrower focuses: Upstart Network mediates Artificial Intelligence (AI)-driven consumer loans via partner banks [120], On Deck Capital specializes in small business financing through brokers and intermediaries [121], and Prosper offers personal loans from retail and institutional investors [122]. Consumer loans have declined for platforms in the USA [123]. Due to the widespread use of credit cards, many borrowers use online lending marketplaces primarily for debt consolidation [88].

A key regulatory development was the passage of the California Commercial Financing Disclosure Law in 2019, implementing mandatory disclosure requirements for small business financing, including the annual percentage rate, to increase transparency and borrower confidence [124]. In addition, the ruling in Madden v. Midland Funding LLC (2015) created legal uncertainty regarding the permissibility of circumventing state usury limits by purchasing bank loans. This led to a decline in marketplace lending and an increase in personal bankruptcies in the affected states [125].

#### Business model development of LendingClub.

LendingClub launched in 2007 as a Facebook app and then as one of the first online lending marketplaces in the USA, mediating loans between retail investors and private borrowers [126]. One year later (2008), it registered its offerings as securities with the Securities and Exchange Commission (SEC) [127]. At LendingClub, the investment amount was distributed in $25 pieces (called notes) across different loan projects, which were grouped into classes A-F, from low to very high risk/return [128]. In 2013, Google acquired shares in LendingClub [129], and LendingClub established its first cooperation with smaller banks, Titan Bank and Congressional Bank [130]. The company has permanently adjusted its offerings and strategy over time. For example, in 2014, it introduced super-prime loans for consumers with excellent credit ratings, zero-interest patient financing, and business lending for SMEs through hedge funds and family offices [131]. The company also went public in the same year. In 2015, the company formed strategic partnerships with Google, Alibaba, and BancAlliance [132], and partnerships with the Opportunity Fund [133] and Sam’s Club [134] to support small businesses. For a simplified investment of online advisors and broker-dealers, “LendingClub Open Integration,” an integration of application programming interface services, was launched [135]. The following year (2016), LendingClub joined forces with competitors Prosper and Funding Circle to found the Marketplace Lending Association, which advocated for responsible business practices in the industry, among other things [136]. It also added auto refinancing to its loan offerings [137]. That same year, LendingClub fired its former CEO, Renaud Laplanche, due to questionable lending practices and a conflict of interest in one of his personal investments, which shook investor confidence and caused its share price to drop [138]. A year later, in 2017, the company launched the mobile iOS application “LendingClub Invest” [139], expanded its investor base to include asset managers and banks [140], and closed its first self-funded securitization deal [141]. In 2018, after a two-year investigation, the Federal Trade Commission (FTC) filed a complaint against LendingClub for failure to comply with FTC and Gramm-Leach-Bliley Act requirements [142]. In the same year, it launched a cooperation with Intuit and Credit Karma to simplify its loan processes and make them more transparent [143]. In response to the FTC complaint, LendingClub changed its website in 2019 [144]. The company also stopped accepting Class E loans [145], emphasized strong institutional investor growth in consumer loans [146], and introduced new investor products [147]. In 2020, LendingClub announced the acquisition of Radius Bank [148], and several adjustments were made due to the COVID-19 pandemic. To minimize overall losses, approval rates for higher-risk borrowers were reduced, applicant screening requirements were increased, and the marketing mix was adjusted [149]. Skip-a-pay was introduced for borrowers, late fees were dropped, interest rates were increased, and the focus was on high-credit quality loans (A and B grade) [150]. At the end of 2020, LendingClub withdrew the notes platform and closed the retail investor business, as the business model was not considered economically viable in the new setting with Radius Bank [151]. The following year (2021), with the completed acquisition by Radius Bank, LendingClub became the first publicly traded U.S. neobank and full-spectrum financial technology marketplace bank [119]. In the process, the yacht lending business was closed, and the auto refinance business was expanded, among other things [152]. In 2022, services such as “Stackit,” a cashback and rewards program for personal borrowers, were introduced [153]. In addition, LendingClub acquired a $1.05 billion personal loan portfolio from MUFG Union Bank, valued at an estimated expense of $4 million [154]. At this time, the Federal Reserve Bank’s interest rate hikes were reflected in lower market revenues at LendingClub. As a result, it reduced its workforce twice in 2023 by 14% each time and announced cost-cutting and reorganization measures to realign its business activities accordingly [155,156]. It also no longer originates new commercial real estate loans [157]. Since its founding (as of March 2024), LendingClub has recorded over 4 million members with over $90 billion in mediated loan volume [158].

**Fig 2:**
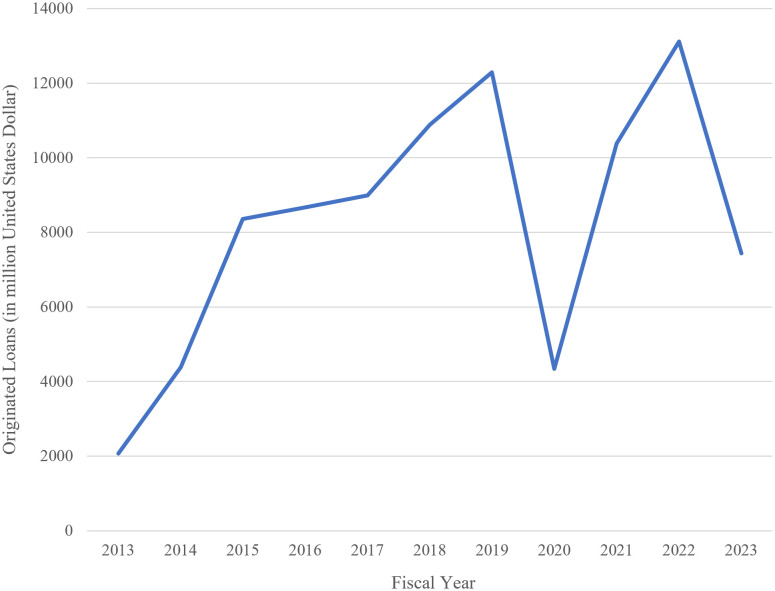
Annual development of originated loans at LendingClub.

The business model development described above is accompanied by the following development of originated loans per fiscal year in Fig 2 (all originated loans from the document archive from LendingClub [159]).

Increasing professionalization (2014), i.e., in addition to the initial public offering, business customers as a new customer segment, and the start of institutional investors, have given the company a significant boost. The increasing offer for both customer sides and the extension of the investor base favored the development of originated loans until 2019. There was a decline in originated loans in 2020. At the same time, various adjustments were made to the loan offering due to the COVID-19 pandemic, and the company specialized in serving institutional investors only. The following two years (2021 and 2022) showed an upswing in originated loans when LendingClub significantly changed its business model with the acquisition of Radius Bank in 2021. However, this was followed by a decline in originated loans as the company had to undergo considerable cost-cutting and reorganization measures in 2023, such as employee redundancies, to adapt to the more difficult market conditions due to higher interest rates.

As of March 2024, LendingClub operates as a bank and lending intermediary. In the lending business, it mediates various loans from institutional investors to private individuals, medical practices (or patients), and business professionals. The various loan types differ especially in loan amount (from $500 to $15 million), term (from 6 months to 25 years), and interest rate (from 0 to 30.99% p.a.). The loan type and conditions depend on the target group, the intended use, and creditworthiness.

Table 2 shows the current business model (as of March 2024) in the lending business in the building blocks of customer segments and value propositions. The banking business is not considered due to the scope of the study.

**Table 2:** Customer segments and value propositions at LendingClub.

	Customer segments	Borrower										Lender
		Private individuals		Medical practices/patients				Business professionals				Institu-tional investors
**Value propositions**	**Loan type**	Personal Loans	Auto Refi-nancing	Patient Solutions (via a registered doctor)				Business Loans				**ND**
				Installment Loans^a^		Revolving Lines of Credit^b^		Small Business Loans^c^	Small Business Administration (SBA)			
									SBA 7(a) Loans	SBA 504 Loans	SBA Express Lines of Credit	
	**Loan range** **(in $)**	1,000–40,000	4,000–55,000	500–35,000	1,000–65,000	499– 32,000	1,000–32,000	5,000–500,000	200,000–5 m	750,000–15 m	Up to 500,000	ND
	**Term (in months)**	24–60	24–84	6–84	6–84	ND	ND	12–60	Up to 25 years	Up to 25 years	ND	ND
	**Interest rates p.a.** **(in %)**	9.57–35.99	4.99–24.99	0	3.99–30.99	0 for 6, 12, 18, or 24 months, then 26.99	17.90 for 24, 36, 48, or 60 months, then 26.99	ND	ND	ND	ND	ND
	**Origi-nation fees** **(in %)**	3–6	ND	ND	ND	ND	ND	ND	ND	ND	ND	ND

a by LendingClub Bank

b by Comenity Capital Bank

c with Accion Opportunity Fund

Note: ND = not disclosed; no reliable publicly available information was identified in the sources analyzed.

### Marketplace lending in Germany: auxmoney

#### Institutional framework and lending landscape.

Germany has a decentralized banking system structured under the three-pillar model, consisting of private commercial banks, public-sector banks, and cooperative banks [70]. Bank financing dominates, while institutional investors such as pension funds and insurers play a minor role. The venture capital market is shaped primarily by international investors [71]. In traditional banking, investments in digitalization focus heavily on security and data protection, with banks increasingly expanding online and mobile services and partnering with or acquiring FinTechs and online lending marketplaces [160].

Germany is part of the eurozone’s Single Supervisory Mechanism (SSM), with the European Central Bank (ECB) overseeing major financial institutions. The ECB supervises 120 major institutions, including Germany’s largest banks. Smaller and medium-sized banks are supervised by the Deutsche Bundesbank and the Federal Financial Supervisory Authority (BaFin), which coordinate closely with the ECB to ensure alignment with EU-wide regulations [161]. Financial supervision also extends to financial service providers such as online lending marketplaces and payment companies, which must comply with capital requirements, risk management, liquidity standards, and reporting obligations [162,163]. Germany follows the BCBS framework [118].

In Germany, online marketplace lending follows the assignment model, where a transaction bank enters a loan agreement with the borrower, disburses the loan, and subsequently assigns the receivable to the lender. The purchase occurs before the loan agreement is concluded [77].

#### Market situation and key players.

Germany is considered the second largest market in Europe, with a market share of 17.7% [164], with auxmoney as the leading online lending marketplace [165] since it replaced the long-standing market leader, smava, in 2015 [166].

The former main players, auxmoney, smava, and Lendico, initially launched with a p2p approach, have changed their business models considerably. auxmoney has relied on institutional investors exclusively since 2022 [167]. smava transformed into a pure loan comparison portal in 2019. Lendico has focused solely on business loans since its sale by Rocket Internet in 2017 [168]. Acquired by ING Diba in 2018, it has been fully integrated into ING Diba’s business banking division for digital SMEs and self-employed lending since 2022 [169].

#### Business model development of auxmoney.

auxmoney began cooperating with SWK Bank in 2008 to process and grant loans between retail investors and private borrowers. Initially criticized for lacking transparency and charging fees before loan conclusion [170], auxmoney introduced major process improvements in 2013 [171], cut the cost of loan applications [172], and introduced mobile crowdfunding and the “auxmoney score,” a credit rating system that improved funding speed [173]. In addition, auxmoney began targeting the self-employed and freelancers with its (personal) loans [174]. In 2014, it lowered the minimum investment to €25 [175], and welcomed family offices as investors. The next year (2015), the first institutional investor, the insurer Aegon, was taken on board, and auxmoney was added to the Finanzcheck.de comparison portal [176]. In 2016, additional institutional investors followed, including ProSiebenSat.1 Media SE [177]. The following year (2017), auxmoney expanded partnerships with retail banks, and the direct bank N24 [178], and introduced digital signatures with Idnow [179]. In 2019, auxmoney extended its borrower base to include businesses, offering loans up to €750,000 through solarisBank [180]. That year also saw partnerships with Miles & More [181], and international offices in Budapest and Málaga, followed by Dublin in 2020 [182]. In 2020, Centerbridge acquired a majority stake, while existing venture capital investors, Index Ventures, Union Square Ventures, and Foundation Capital, stayed on board [183]. The same year, auxmoney entered a financing partnership with BNP Paribas [184], began investing on its own online lending marketplace [185], and founded auxmoney Investments to manage funding structures [186]. auxmoney was aimed specifically at global institutional investors [187]. In 2021, new investors, including Citigroup, Chenavari [185], and Citi [188] joined, and the first ABS was issued [189]. During the year, auxmoney rebounded from COVID-19 with 113% loan growth and 41% revenue growth [190]. In 2022, it issued its second ABS [191], gained Natixis as an investor [188], and closed its platform to retail investors [167]. A year later (2023), a third ABS followed [192], alongside a majority stake acquisition of leading Dutch competitor Lender & Spender BV [193], indicating consolidation in Germany’s FinTech market [194]. By 2024, auxmoney had placed four social ABS bonds totaling €1.3 billion [195], and claims to be Europe’s leading digital platform for personal loans [196]. Since its founding (as of March 2024), auxmoney has mediated nearly 600,000 loans over €4.5 billion [197].

**Fig 3:**
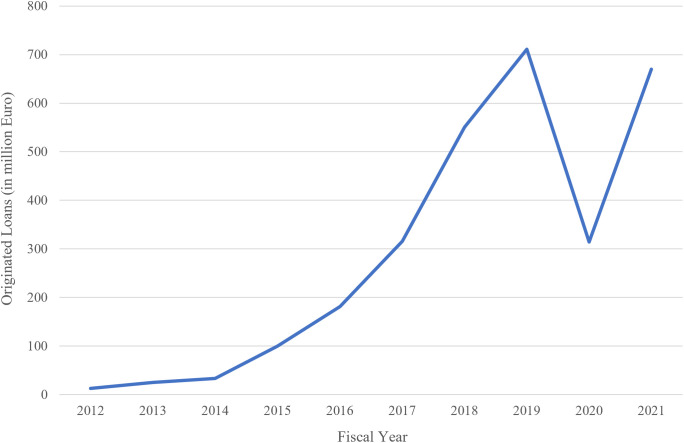
Annual development of originated loans at auxmoney.

The development of the business model described above is accompanied by the following development of originated loans per fiscal year in Fig 3 (all originated loans from the auxmoney press archive [198], and after consultation with auxmoney).

Following its expansion to the self-employed and freelancers as borrowers (2013), family offices (2014), and institutional investors (2015), originated loan volumes rose significantly. A temporary business loan offer in 2019 and the international expansion in 2019–2020 were followed by a pandemic-related decline in 2020, despite strategic investments and partnerships. From 2021, ABS issuances and investor growth marked a new scaling phase although auxmoney no longer reports any new loan volumes.

As of March 2024, auxmoney mediates loans from institutional investors and auxmoney itself to private individuals, SMEs, the self-employed, freelancers, and founders. All apply for the same personal loan product (from €1,000 to €50,000, from 12 to 84 months), with conditions depending on the intended use and creditworthiness.

Table 3 shows the current business model (as of March 2024) in the building blocks of customer segments and value propositions.

**Table 3:** Customer segments and value propositions at auxmoney.

	Customer segments	Borrower			Lender
		Private individuals	SME	Self-employed, freelancers, and founders	Institutional investors
**Value propositions**	**Loan type**	Personal Loans	Corporate Loans (not a separate product, it’s personal loans)		ND
	**Loan range** **(in €)**	1,000–50,000	1,000–50,000		ND
	**Term** **(in months)**	12–84	12–84		ND
	**Interest rates p.a. (in %)**	ND	ND		ND
	**Fees**	Ø 3.5% of the net loan amount	Ø 3.5% of the net loan amount		ND

Note: ND = not disclosed; no reliable publicly available information was identified in the sources analyzed.

## Cross-case analysis

### Institutional framework and lending landscape

While the UK, the USA, and Germany follow the BCBS framework, their banking structures, regulatory approaches, and lending models differ. The UK operates a centralized banking system with London as a global financial hub, the USA follows a decentralized structure with twelve Federal Reserve Banks, and Germany adheres to a three-pillar banking model, where public and cooperative banks play a major role [70]. Capital markets are most developed in the USA and the UK, supporting active institutional investor participation, while Germany’s more conservative market has slowed this transition. Banks in all three countries increasingly act as marketplace investors, driven by regulatory requirements (e.g., Environmental, Social, and Governance [ESG] criteria) and access to new borrower segments. Some platforms, like LendingClub (the USA) and Zopa (the UK), acquired banking licenses, while others, such as Lendico, have been absorbed into traditional banks such as ING-DiBa (Germany).

Despite the alignment with BCBS standards, supervisory intensity varies. Germany’s EU-based oversight is considered stricter than in the UK or the USA, particularly regarding capital requirements [199], whereas the more liberal USA approach contributes to the global reach of its investment banks [200]. Regulatory tightening across all countries has improved transparency and investor protection, but limited retail investor participation. Alongside advances in data analytics and credit scoring, this has driven the professionalization and scaling of marketplaces, making them more attractive to institutional investors and accelerating the transition from p2p to peer-to-institution (p2i) models.

The lending models also reflect these national differences. In the UK, genuine p2p lending is permitted, in the USA, a debt-dependent notes model with partner banks is used, and in Germany, an assignment model with fronting banks [77]. Despite these structural differences, all three countries maintain a comparable regulatory focus through international coordination.

### Market situation and key players

The online lending marketplace industry has matured significantly in all three countries, although there are signs of consolidation and a slowdown in growth momentum. The UK market remains highly fragmented but is dominated by Funding Circle, which holds almost half the market share despite growing competition and declining active marketplace numbers. In the USA, LendingClub leads a smaller group of major players and has evolved into a full-service marketplace bank. In Germany, the market is more consolidated, with auxmoney becoming the dominant marketplace after competitors such as smava and Lendico modified or abandoned their original models.

Overall, the online lending market is becoming more concentrated in each country, with the leading marketplaces moving away from p2p lending and increasingly focusing on institutional investors or positioning themselves as banks.

### Business model development

All three marketplaces began with a p2p model, with retail investors funding borrower groups unserved by traditional banks. While auxmoney and LendingClub initially focused on consumer loans, Funding Circle positioned itself as a provider of SME loans from the outset. Over time, all three marketplaces integrated institutional investors. Responding to increasing regulatory requirements, the growing importance of larger transaction volumes, and the need for more professional risk management and transparency, they progressively phased out access for retail investors and increasingly shifted toward serving more demanding and sophisticated customer groups on the investment side.

**Fig 4:**
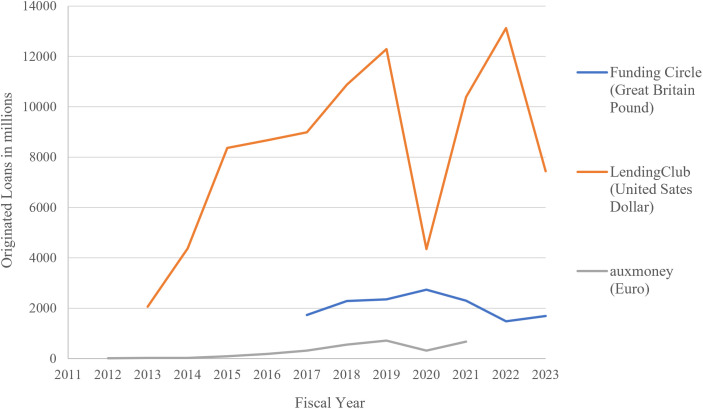
Annual development of originated loans in comparison.

The development of the business models described above, focusing on shifting customer segments, is accompanied by the following development of originated loans per fiscal year across all three marketplaces, as shown in Fig 4 (for a more detailed view of the figures and data, see the single cases).

All three marketplaces significantly increased their originated loans after onboarding institutional investors: Funding Circle in 2011, LendingClub in 2014, and auxmoney in 2015. Funding Circle and auxmoney showed notable growth after closing their retail investment businesses (2022 and 2021), while LendingClub’s development reflects this shift (2020) and its subsequent integration of banking operations (2021). In both the UK and the USA, government-backed loan programs have further strengthened institutional participation. Funding Circle is part of the UK’s Recovery Loan Scheme and holds an SBA license in the USA, while LendingClub offers SBA 7(a), SBA 504, and SBA Express credit lines under U.S. federal programs.

The expansion of the borrower segments followed a differentiated path. LendingClub’s loan volumes rose as it broadened access to new customer types in 2014, while auxmoney temporarily saw a decline when adding business loans in 2019 and discontinued the offering shortly after. However, loans for the self-employed have been available as personal loans since 2013 at auxmoney. Funding Circle has consistently focused on SME lending.

Table 4 shows the customer segments currently addressed by the three market leaders (as of March 2024).

**Table 4:** Customer segments in comparison.

Customer segments	Borrower					Lender	
	Private individuals	SME	Self-employed, freelancers, and founders	Medical practices/patients	Further business professionals	Retail investors	Institutional investors
**Auxmoney**	X	X	X	–	–	Closed 2021	X (since 2015, since 2014 family offices)
**Funding Circle**	–	X	X	–	–	Closed 2022	X (since 2011)
**LendingClub**	X	X	X	X	X	Closed 2020	X (since 2014)

X = applies

- = does not apply

All three marketplaces now focus exclusively on institutional investors and offer loans to SMEs, the self-employed, freelancers, and founders. Although auxmoney also offers personal loans, LendingClub addresses a much broader customer base, including medical practices/patients, business professionals, and diverse bank customers, after it acquired Radius Bank in 2021.

For further analysis, Table 5 presents the three marketplaces’ value propositions aligned to the shared customer segment (SME, the self-employed, freelancers, and founders) (as of March 2024).

**Table 5:** Value propositions in comparison.

	Funding Circle				LendingClub				auxmoney
**Loan type**	Business Loans*	Government-Backed Recovery Loans (Recovery Loan Scheme)	Asset Finance	FlexiPay	Small Business Loans	SBA 7(a) Loans,	SBA 504 Loans	SBA Express Lines of Credit	Corporate Loans (not a separate product, it’s personal loans)
**Loan range**	£10,000–£500,000	£25,001–£250,000	£10,000–£5 m	£1,000–£250,000	$5,000–$500,000	$200,000–$5 m	$750,000–$15 m	Up to $500,000	€1,000–€50,000
**Term**	6–72 months	6–72 months	6–84 months	3 months	12–60 months	Up to 25 years	Up to 25 years	ND	12–84 months

The comparison reveals that auxmoney offers the smallest loan range (€1,000–€50,000), Funding Circle a much wider (£1,000–£5 million), and LendingClub the widest ($5,000–$15 million). Terms are relatively similar for auxmoney (12–84 months) and Funding Circle (3–84 months), whereas LendingClub offers much longer terms (12 months–25 years), reflecting its broader loan spectrum. Interest rates and fees vary widely by country and lender and are therefore not included in this comparison. The value propositions reflect the strategic positioning and structural differences between national markets.

## Discussion

### Interpretation of results

Our analysis shows an increasing convergence of the central business model components “customer segments” and “value propositions” of all three marketplaces. Despite different national contexts and original orientations, Funding Circle, LendingClub, and auxmoney have evolved from p2p models with retail investors to professionally managed, institutionally funded marketplaces. While the customer segments differ in detail, all three now focus on previously underserved borrower groups, especially SMEs, the self-employed, freelancers, and founders. At the same time, the market has consolidated. A few large platforms now dominate in every country, while smaller competitors have exited the market or fundamentally changed their business models. This development may mark a turning point, indicating the emergence of a dominant design in business model components of online lending marketplaces.

This change was driven by increasing regulatory requirements, particularly regarding investor protection and transparency, as well as technological advances in credit scoring and data analysis. This interpretation is consistent with prior research showing that borrower information remains difficult to assess, that voluntary disclosure may be strategically used by borrowers, and that expert investors can make better use of available borrower and loan information than nonexpert investors [30,33]. In this context, all three marketplaces excluded retail investors between 2020 and 2022. Institutional capital has become the new standard, enabling scalability and aligning with regulatory expectations.

Capital market structures also influenced investor engagement. Institutional participation gained momentum in the USA and the UK due to more developed markets, while the conservative environment in Germany slowed this shift. Banks also became marketplace investors to mitigate disruption and meet (i.e., social) ESG targets. Thus, they assume a dual role by shaping market conditions externally as incumbents while participating directly in the marketplace model. Public lending programs reinforced this trend: Funding Circle participates in the UK Recovery Loan Scheme and holds a U.S. SBA license, while LendingClub offers SBA-backed loans under federal programs. The marketplaces now act as financial intermediaries and policy instruments for targeted SME lending. However, the exclusion of retail investors limits broader participation in alternative finance.

External and investor-driven changes shape business model convergence and reinforce an emerging upmarket pattern: a gradual move toward more profitable and demanding customer groups. All three marketplaces progressively moved away from fragmented retail investor bases toward institutional investors with higher expectations for professionalism and transparency. For instance, LendingClub acquired a banking license to strengthen its service portfolio, Funding Circle expanded and professionalized its SME lending operations under regulatory frameworks, and auxmoney implemented securitization structures to attract institutional capital. In this evolution, online lending marketplaces professionalized their operations and increasingly adopted elements of incumbent banking models, such as regulatory compliance, standardized risk management, and banking licenses. This development is consistent with early analytical projections about the disruptive potential of online lending marketplaces [2]. According to these authors, the disruptive potential of online lending marketplaces varies depending on the market side, with the investors’ side showing a lower disruptive potential than the borrowers’ side, and therefore lowering the overall disruptive potential of online lending marketplaces compared to established banks [2].

Borrower segments have evolved differently across the three marketplaces. Funding Circle has consistently focused on SMEs, reflecting the structural weaknesses in UK banks’ lending to small businesses. LendingClub has expanded its borrower base to include private customers, medical practices, tradespeople, and bank customers since the acquisition of Radius Bank. auxmoney, on the other hand, briefly introduced business loans but discontinued its offering. Today, it targets the SME segment with personal loans. Despite national differences, the core segments of borrowers on the marketplaces overlap.

The marketplaces converge into a dominant configuration: digital, scalable, and institutionally supported lending models serving underserved borrower groups. While some local differences remain, structurally similar components have emerged internationally.

### Generic model for dominant business model components

**Fig 5:**
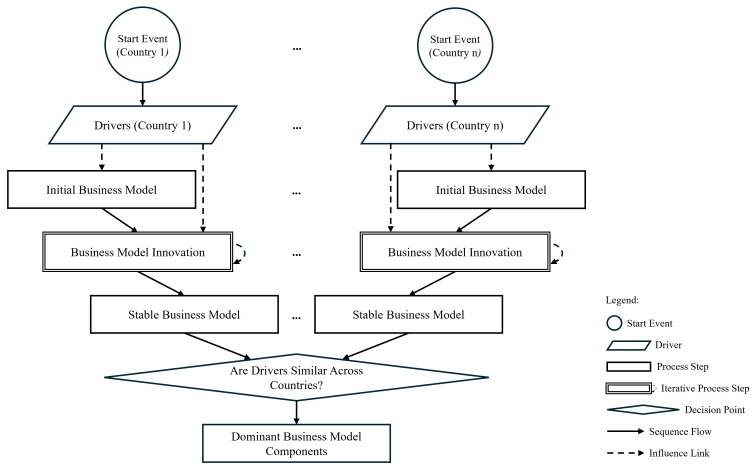
Generic model of international dominant business model component emergence.

Based on our analysis, we propose a generic, process-oriented model explaining how internationally dominant business model components can emerge in potentially disruptive two-sided platform markets. The model illustrates how diverse initial national business models undergo adaptation through external drivers and iterative innovation, leading to the international convergence of specific business model components (Fig 5). It is based on the case analysis of Funding Circle (the UK), LendingClub (the USA), and auxmoney (Germany) and integrates theoretical concepts from the business model, platform, and disruptive innovation literature (e.g., [35,40,56,50]). The focus is on the customer-centric building blocks “customer segments” and “value propositions,” whose international convergence signals the emergence of dominant components.

The process begins with *Start Events* that describe the initial contextual conditions in each country and favor the market entry of potentially disruptive marketplace players. Examples include technological change (e.g., Web 2.0), social openness to new financial intermediaries (the UK), declining trust in banks (the USA), or structural gaps in retail banking (Germany). These conditions led to country-specific *Drivers* such as regulatory changes, technological advances, and market dynamics, which often influence and reinforce each other. Funding Circle, for example, benefited from the FCA’s regulatory framework, while LendingClub illustrates the early adoption of Web-based technologies with its Facebook app, and auxmoney was strongly influenced by cooperation constraints with partner banks.

Based on this, *Initial Business Models* were created that varied greatly between countries and within a country. This heterogeneity reflected different regulatory frameworks and uncertain assumptions about customer needs. While auxmoney and LendingClub initially focused on p2p financing between retail investors and private borrowers, Funding Circle addressed SMEs at an early stage. The variation corresponds to the early phase in the dominant design theory [24,25] and is consistent with the theory of disruptive innovation, where entrants initially serve underserved or previously untapped customer segments [49,50,201].

Over time, marketplaces adapted their business models to increasingly clear customer needs and to their value network [14–18]. In addition to refining their fit with customer needs, the marketplaces exhibited an emerging upmarket pattern [50], primarily by shifting toward more attractive institutional investor segments, while continuing to target underserved borrower groups. This process of *Business Model Innovation* is iterative and largely driven by external influences. Technological advances in data analysis and scoring have improved risk management. Regulatory requirements such as increased transparency obligations and investor protection measures complicated the involvement of retail investors. At the same time, increasing competition, in which banks also became actively involved, increased the pressure to professionalize. The marketplaces responded by, e.g., auxmoney implementing ABS structures and extending its range of loans, LendingClub expanding its medical financing and acquiring a banking license, and Funding Circle differentiating its SME portfolio. During this development, all three marketplaces ended their retail investor business and switched entirely to institutional investors.

This process continues until a temporarily dominant business model configuration emerges that serves most customers, becoming established as a *Stable Business Model* in the national context. While prior studies suggest that a uniform configuration tends to prevail within industries over time [13,14,23], Pur et al. [10] cannot confirm this for the German online lending market. Due to the specific competitive situation, the business models of the market leaders are strongly differentiated.

If similar external drivers act in different countries, this can lead to the development of *Dominant Business Model Components* at an international level. The cases examined show corresponding convergences that indicate such an emergence. While Funding Circle continues to focus exclusively on SME loans, which is the most underserved borrower group in the UK, auxmoney combines loans for SMEs and private borrowers, and LendingClub has expanded its model to include banking services after the acquisition of Radius Bank. Despite these partially local differences in customer segments and value propositions, structural similarities exist. All three marketplaces now focus exclusively on institutional investors and serve SMEs as borrowers. This convergence of business model components supports theoretical perspectives on business model standardization and the emergence of dominant configurations in an international context [13,14,23].

### Theoretical contributions

This study advances theoretical understanding in three key areas: the extension of the dominant design theory to the business model level, the emergence of component-level business model convergence in international platform markets, and the role of external drivers in shaping business model innovation across countries.

First, we contribute to the literature on dominant designs by extending this concept beyond its traditional technology- or architecture-based perspective [10,25,26] to the business model level. Our findings show that internationally dominant business model components can emerge in potentially disruptive two-sided platform markets, where convergence is driven by comparable external forces such as regulatory changes, technological advances, and market dynamics. Instead of full business model standardization, we observe convergence at the level of the customer-oriented components, customer segments and value propositions across different national contexts. Furthermore, by applying dominant design theory in a regulated service industry, this study extends its conceptual scope beyond the predominantly technological contexts addressed in prior research [25].

Second, this study extends existing research on platform business model evolution [27] and standardization processes in digital platform markets, which have focused primarily on technological, organizational, or regulatory dimensions [28]. It also complements research on online lending marketplaces that has examined information asymmetries, signaling, voluntary disclosure, investor behavior, geographical frictions, and platform monitoring at the level of lending and investment decisions as well as platform risks [29–34]. In this context, our paper shows possible reactions of online lending marketplaces when information asymmetries prevail. By identifying the convergence of business model components shaped by external forces, we broaden the understanding of how platform ecosystems evolve under comparable conditions. While previous studies discuss the emergence of uniform business model configurations over time [13,14,23], and recent research highlights national differentiation due to competition and multi-homing [10], our findings suggest that component-level standardization can occur internationally, particularly when similar market conditions prevail and market leaders operate in parallel rather than in direct competition.

Third, we advance research on external drivers of business model innovation [22] by demonstrating how regulatory changes, technological advances, shifting customer preferences, and systemic competitive pressures can interact to generate convergent patterns of business model development across countries.

### Practical implications

Our results show several practical implications for policymakers, banks, FinTechs, online lending marketplace operators, institutional and retail investors, and borrowers.

Policymakers have contributed significantly to the shift from p2p to p2i lending models, particularly through stricter investor protection regulations. A better understanding of convergence mechanisms can support the development of future regulations that balance innovation, competition, and financial stability, including regarding global sustainability goals and ESG criteria.

Banks are no longer just observers, but investors, partners, and competitors. As institutional investors, banks benefit not only from strategic access to online lending marketplaces but also from aligning with ESG-oriented regulations that position these investments as part of a broader sustainability agenda. At the same time, they must reposition themselves in a financial ecosystem where online lending marketplaces are increasingly becoming banks.

FinTechs and marketplace operators must adapt to institutional standards in the areas of risk assessment, transparency, and regulatory compliance while maintaining national differentiation. At the same time, the movement of online lending marketplaces into the main market of traditional banks is once again leaving a gap in the lower market segment, opening opportunities for niche providers, social lending models, and new disruptive players. Entrant firms with marketplace business models could learn from the theoretical path we described here, especially when business models are developed strategically. Being aware of arising upmarket and regulatory pressures on initial business models with disruptive potential originating from the low end of both market sides creates clear pathways and guidance for the business model convergence of the future state.

Institutional investors benefit from professionalized, standardized marketplaces, which enable larger investments, stable returns, and manageable risks. While institutional capital improves access to credit for underserved groups, excluding retail investors increasingly limits opportunities for broader financial participation.

Borrowers benefit from larger loan volumes, improved scoring systems, and more transparent conditions. As marketplaces professionalize and expand their offerings, underserved segments, such as SMEs and the self-employed, gain better access to credit.

## Conclusion

### Summary of key findings

Online lending marketplaces have developed into a potentially disruptive force in the global banking industry. As in other industries, it was expected that a dominant business model configuration would emerge among successful marketplaces operating beyond niche segments [13,14,23]. Our analysis of market leaders in key growth markets where the industry originated (the UK, the USA, and Germany) reveals a clear convergence in the key business model components, customer segments and value propositions, which are considered critical for customer-centric business model innovation [41] and product-market fit [36]. This convergence occurs independently across national contexts under comparable regulatory frameworks, despite differences in supervisory models, market structures, and intermediation mechanisms. While local distinctions remain, structurally similar business model components have emerged internationally, indicating the emergence of a dominant design at the component level.

All three market leaders examined, Funding Circle (the UK), LendingClub (the USA), and auxmoney (Germany), increasingly aligned their business models around the same core segments after years of iterative innovation shaped by regulatory tightening, technological advances, and changing market structures. Today, all three serve SMEs, the self-employed, freelancers, and founders, and rely entirely on institutional capital. The convergence observed is not driven by direct competition but by parallel responses to similar external pressures. Whereas seminal articles on information asymmetries examined signaling, voluntary disclosure, investor behavior, geographical frictions, and platform monitoring at the level of lending and investment decisions as well as platform risks [29–34], our paper shows how these mechanisms translate into business model development and convergence at the platform and industry level.

While national convergence was not observed due to competitive dynamics and multi-homing [10], our findings reveal a clear alignment of dominant business model components across national contexts. This convergence is accompanied by increasing market concentration, with a few dominant platforms prevailing in each country. This contributes to advancing theories on dominant design and business model standardization in international platform markets [14,25,26].

Based on these findings, we propose a generic model for the emergence of internationally dominant business model components in potentially disruptive two-sided platform markets. The model highlights the central role of external forces in shaping convergence and addresses research gaps by extending dominant design theory to business models, analyzing external pressures as drivers of innovation, and illustrating how convergence can occur despite national fragmentation.

### Limitations and further research

This study has several methodological, conceptual, and data-specific limitations that also point to directions for further research.

As a qualitative case study, the findings are not directly transferable to other platform markets or industries. The analysis focuses on three leading online lending marketplaces in developed economies (the UK, the USA, and Germany). Future research should test the proposed generic model in other geographic contexts, particularly in emerging markets, and under different regulatory or economic conditions. Application to other platform types could reveal whether internationally dominant business model components are also emerging beyond the financial sector. Quantitative studies may help empirically validate the model and its underlying assumptions.

While this study emphasizes the convergence of business model components, it does not address the transition from product to process innovation, a central element in dominant design theory. Further research could investigate whether similar mechanisms apply in other service-oriented platform markets and identify appropriate indicators for these transitions.

Macroeconomic and cultural factors, such as interest rate policy or national financial preferences, were only indirectly considered in our analysis, yet they may significantly influence business model innovations. Extending the model to include such structural or contextual variables could enhance its explanatory power and applicability across different platform contexts.

Finally, the reliance on publicly available secondary data limits insight into online lending marketplaces’ strategic decision-making processes. Future studies should integrate primary data sources, such as expert interviews with marketplace operators, regulators, or institutional investors, to better understand business model development and strategic adaptation.
